# Supplementary material for: The histone demethylase Phf2 acts as a molecular checkpoint to prevent NAFLD progression during obesity
Source: Nat Commun. 2018 May 29;9:2092. doi: 10.1038/s41467-018-04361-y (PMC5974278; doi:10.1038/s41467-018-04361-y)
Supplement: Supplementary file 1 — Supplementary Information [file 41467_2018_4361_MOESM1_ESM.pdf]

The histone demethylase Phf2 acts as a molecular checkpoint to prevent NAFLD progression during obesity

Julien Bricambert[1, 2, 3], Marie-Clotilde Alves-Guerra[1, 2, 3], Pauline Esteves[1, 2, 3], Carina Prip-Buus[1, 2, 3], Justine Bertrand-Michel[4], Hervé Guillou[5], Christopher J. Chang[6, 7], Mark N. Vander Wal[6], François Canonne-Hergaux[9, 10, 11], Philippe Mathurin[12], Violeta Raverdy[13], François Pattou[13], Jean Girard[1, 2, 3], Catherine Postic[1, 2, 3] and Renaud Dentin[1, 2, 3*]

[1] INSERM, U1016, Institut Cochin, Paris, France.

[2] CNRS, UMR8104, Paris, France.

[3] Université Paris Descartes, Sorbonne Paris Cité, Paris, France.

[4] Plateau de lipidomique, Bio-Medical Federative Research Institute of Toulouse, INSERM, Plateforme MetaToul, Toulouse, France.

[5] INRA-ToxAlim, Toxicologie Intégrative et Métabolisme, Toulouse, France.

[6] Department of Chemistry and Molecular and Cell Biology, University of California, Berkeley, California, 94720, United States.

[7] Howard Hughes Medical Institute, University of California, Berkeley, California, 94720, United States.

[9] INSERM U1043-CPTP, Toulouse, F-31300, France;

[10] CNRS, U5282, Toulouse, F-31300, France;

[11] Université de Toulouse, UPS, Centre de Physiopathologie de Toulouse Purpan (CPTP), Toulouse, F-31300, France

[12] Department of Hepatology, Lille University Hospital, Lille, France; Inserm U 995, Lille.

[13] INSERM, U859 Biotherapies for Diabetes, Lille, France. European Genomic Institute for Diabetes, Lille University, Lille, France. Department of Endocrine Surgery, Lille University Hospital, Lille, France.

*Address correspondence to: Renaud Dentin, Institut Cochin, Département d'Endocrinologie, Métabolisme et Diabète, Faculté de médecine 3ème étage, 24 Rue du Faubourg Saint Jacques, 75014 Paris, France. Phone : 33-1-53-73-27-20; Fax: 33-1-53-73-27-03 ; E-mail: renaud.dentin@inserm.fr

Supplementary Figures

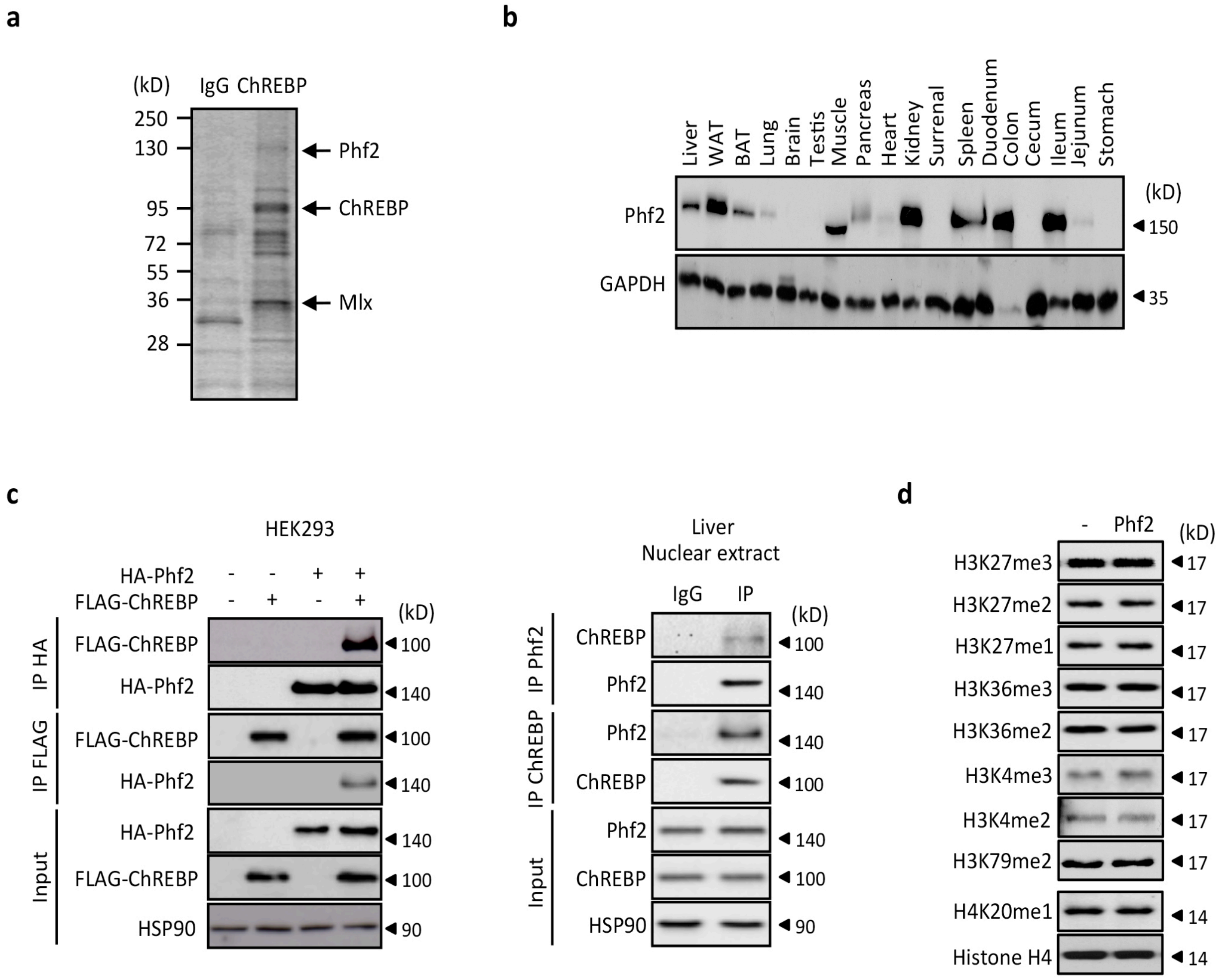

Supplementary Fig. 1 Characterization of Phf2 histone demethylase activity. **a** Biochemical identification of new transcriptional co-regulators for the transcription factor ChREBP from primary cultured hepatocytes incubated with 100 nM insulin and 25 mM glucose for 24 h. ChREBP was immunoprecipitated from nuclear fractions and ChREBP-purified complexes were analyzed by using GC-MS analysis. **b** Phf2 tissue distribution in C57BL/6J mice by immunoblotting. **c** Co-immunoprecipitation experiments between ChREBP and Phf2. (Left) FLAG-tagged ChREBP and HA-tagged Phf2 were overexpressed in 293T cells. CoIP experiments were performed using indicated antibodies (n = 3). (Right) *In vivo* CoIP experiments were performed using indicated antibodies and liver nuclear extracts from fed C57Bl/6J mice (n = 3). **d** Demethylase activity of FLAG-tagged Phf2 immunoprecipitated from 293T cell lysates was assessed using recombinant methylated histones as substrates (n = 3).

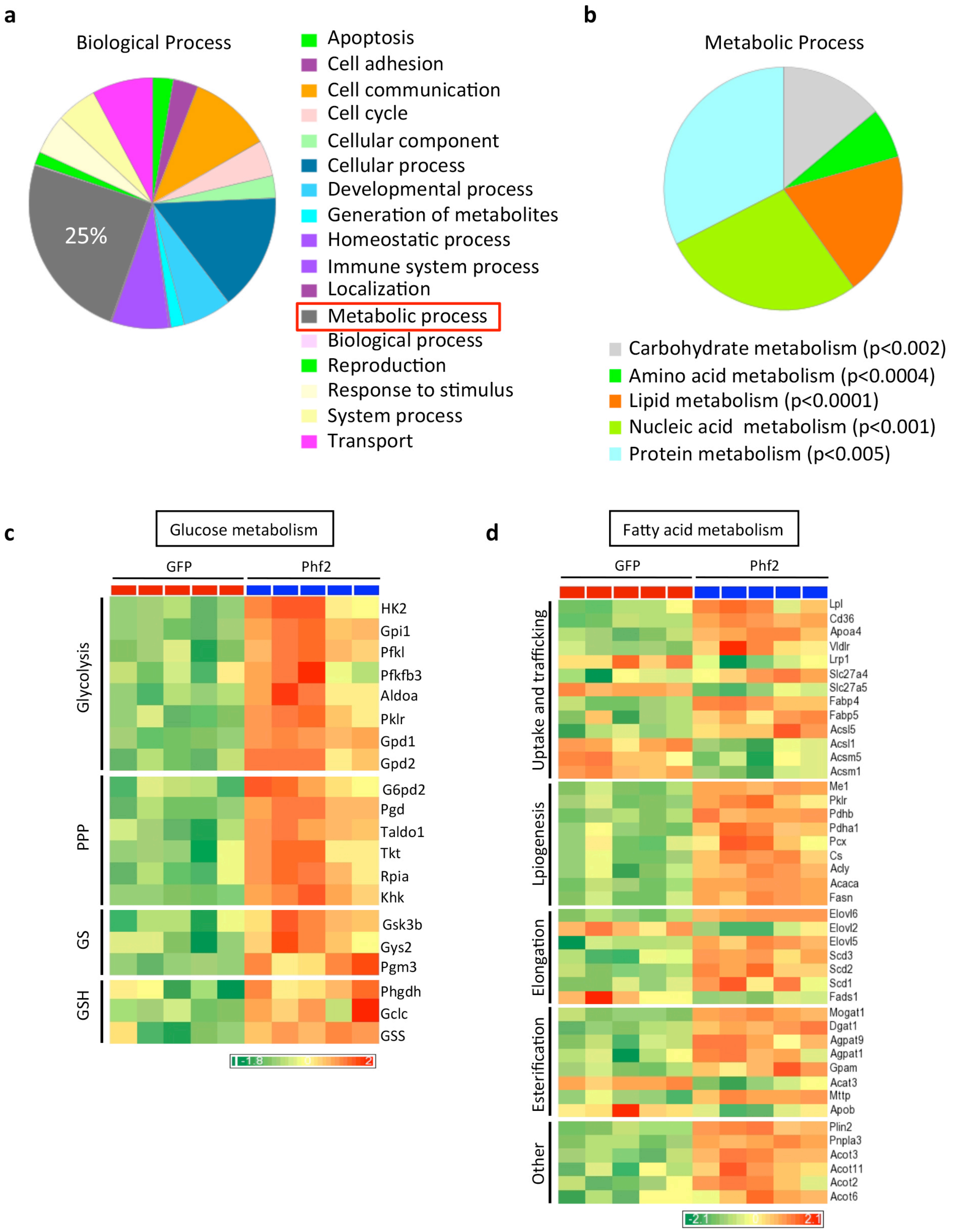

Supplementary Fig. 2 Characterization of Phf2 transcriptional network. GFP or Phf2 were overexpressed for 3 weeks in the liver of C57BL/6J mice through adenoviral-gene delivery and microarray analysis was conducted. Phf2 overexpression resulted in the differential regulation of a significant proportion of genes (921, fold ± 1.5 and $P < 0.05$) involved in several metabolic processes compared to GFP mice. **a,b** Genes that were differentially regulated in the liver of Phf2 mice were subjected to gene ontology enrichment analysis. Pie charts represent step-wise hierarchical tree drill down of significantly enriched terms under the heading biological process and metabolic process sub-category. All enrichment *P* values < 0.05 are indicated in parentheses. **c,d** Heat map visualization of glucose and lipid metabolism between GFP and Phf2 mice (n = 10 per group).

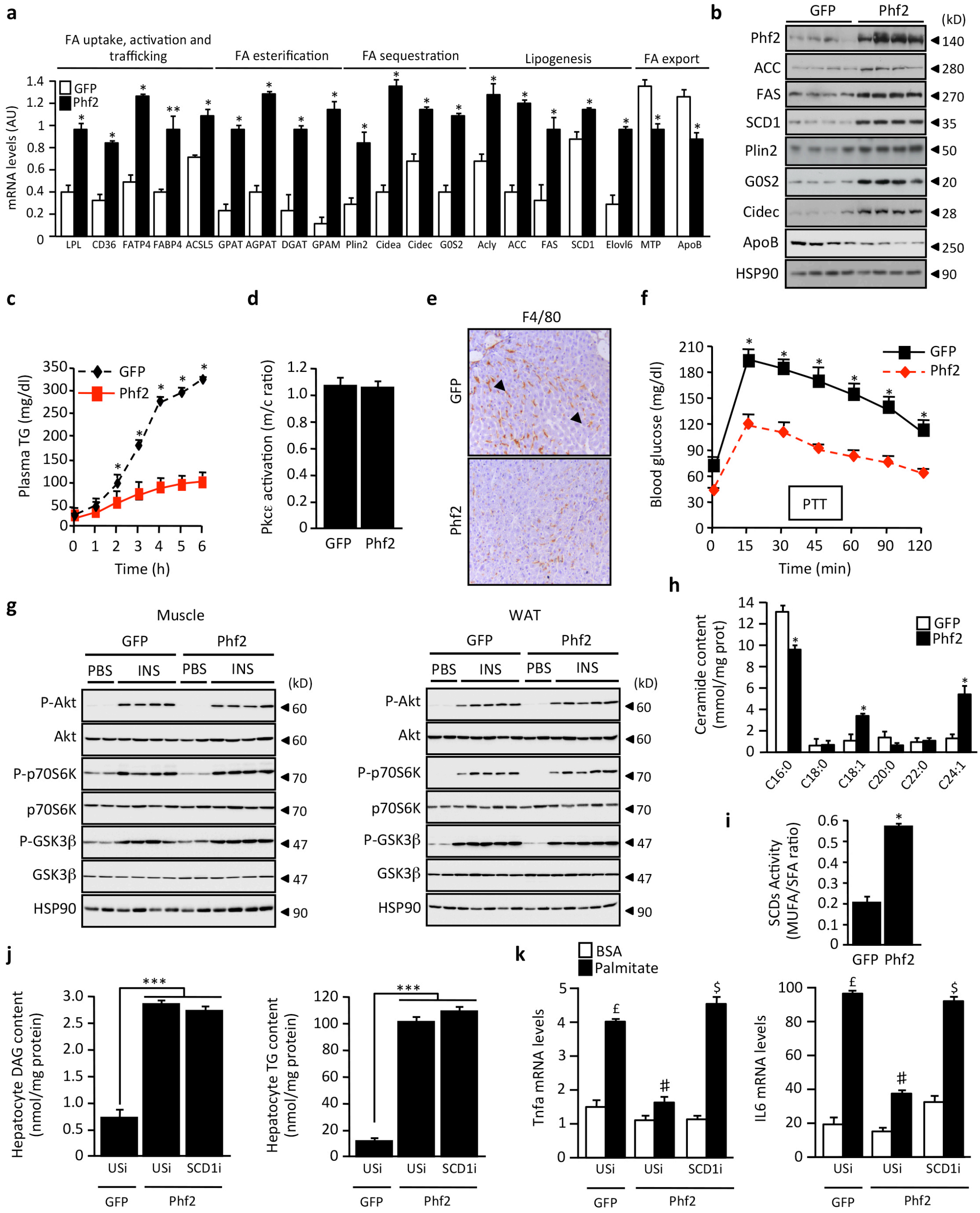

Supplementary Fig. 3 Molecular basis of Phf2-driven hepatosteatosis development. **(a-h)** Mice, injected with either GFP or Phf2 overexpressing adenovirus, were studied 3 weeks later in the fed state. **a** Expression of genes involved in FA metabolism (*n* = 20 per group). **b** Western blot analysis of liver lysates against proteins involved in FA metabolism (*n* = 10 per group). **c** Hepatic TG secretion rates evaluated by measuring plasma TG concentrations in a time course after blocking its turnover with tyloxapol (*n* = 6 per group). **d** Results from the PKCε activation assay (*n* = 8 per group). m/c, membrane compared to cytosolic PKCε protein concentration. **e** Visualization of macrophage infiltration by immunostaining of liver sections with F4/80 antibody. Magnification x200 (*n* = 5 per group). **f** Oral pyruvate tolerance test (*n* = 10 per group). **g** Western blot analysis of the PI3K/Akt signaling pathway in white adipose tissue and skeletal muscle (*n* = 10 per group). **h** MUFA/SFA ratio reflecting SCD1 activity in liver samples (*n* = 10 per group). **(i-j)** Isolated primary cultured hepatocytes overexpressing Phf2 and in which SCD1 expression was inhibited were incubated in the presence of palmitate (480 μM) for 24 h. **i** Hepatocyte DAG and TG content (*n* = 3). **j** Relative expression of Tnfa and IL6 (*n* = 3). All error bars represent mean ± SEM. Statistical analyses were made using unpaired *t*-test **(a,c,d,f,h,i)** or Anova, followed by Bonferonni's test (**j,k**). *$P < 0.01$ GFP compared to Phf2, **$P < 0.05$ GFP compared to Phf2, *** $P < 0.01$ GFP compared to Phf2, £ $P < 0.01$ GFP/USi BSA compared to GFP/USi palmitate, # $P < 0.01$ GFP/USi palmitate compared to Phf2/USi palmitate, $ $P < 0.01$ Phf2/USi palmitate compared to Phf2/SCD1i palmitate.

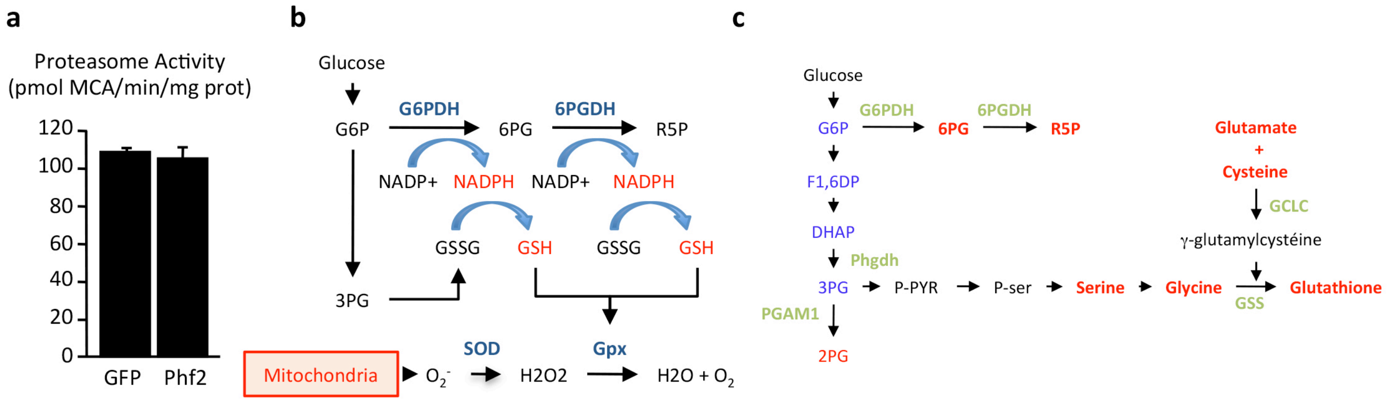

Supplementary Fig. 4. Phf2 diverts glucose fluxes to protect liver from oxidative stress. **a** Measurement of the proteasome activity (*n* = 12 mice per group). **b** Schematic representation of the role of mitochondrial oxidative capacity and glucose metabolism in the regulation of oxidative stress. Increased mitochondrial glucose and fatty acid oxidation is able to generate the superoxide anion radical, due to electron leakage from complexes I and III of the mitochondrial respiration chain. This radical is dismutated by superoxide dismutase (SOD) into hydrogen peroxide (H_2O_2), which is finally detoxified into water by glutathione peroxidase (GPx) and reduced glutathione (GSH). In addition, enhanced glucose flux into the pentose phosphate pathway is able to generate NADPH, which is required to increase GPx activity for H_2O_2 detoxification. **c** Contribution of Phf2 overexpression to liver metabolic reprogramming based on gene expression data set and metabolomic analysis. Genes that are increased in response to Phf2 overerexpression are indicated in green. Metabolic intermediates that accumulate in the liver of Phf2 mice are indicated in red and the ones that are decreased are indicated in blue. All error bars represent mean ± SEM. Statistical analyses were made using unpaired *t*-test (**a**).

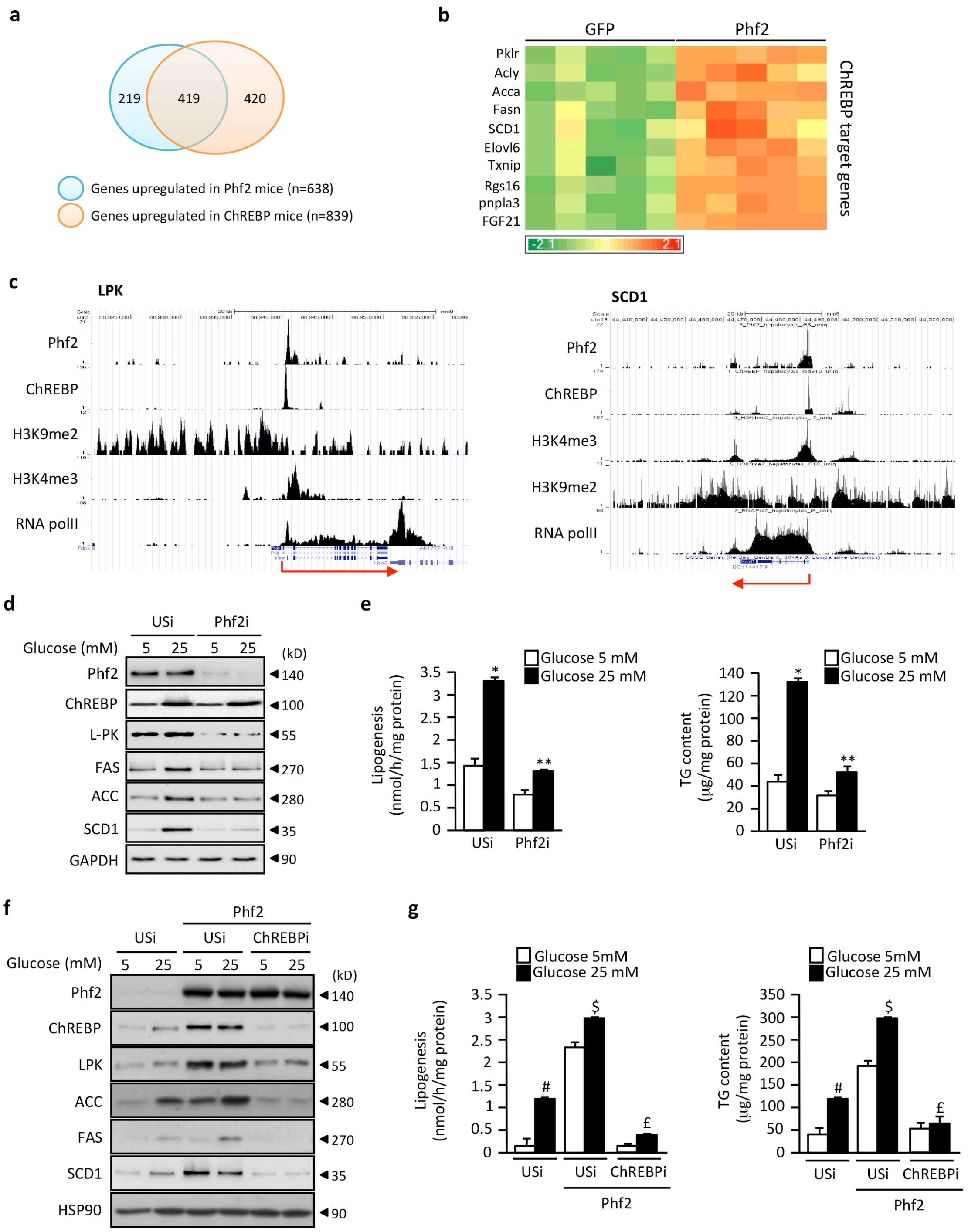

Supplementary Fig. 5. Phf2 acts as a new ChREBP transcriptional co-activator. **a** Venn diagram showing the overlap between genes that are up regulated by Phf2 or ChREBP overexpression in the liver of C57BL/6J mice (fold ± 1.5 and *P* < 0.05). **b** Heat map of known ChREBP upregulated genes from GFP and Phf2 mice (*n* = 5 per group). **c** UCSC genome browser image illustrating normalized tag counts for Phf2, ChREBP, H3K9me2, H3K4me3 and RNA polII at the promoter of known ChREBP-regulated genes (LPK and SCD1) from cultured hepatocytes. **(d,e)** Primary cultured hepatocytes, in which Phf2 expression was inhibited, were incubated 24h with either 5 or 25 mM glucose. **d** Western blot analysis of glycolytic and lipogenic enzymes shown (*n* = 5). **e** *De novo* lipogenic rate, measured by tracing newly synthesized lipids from ^{14}C-glucose for 2 h and total TG contents shown (*n* = 5). **(f,g)** ChREBP expression was inhibited in primary cultured hepatocytes overexpressing Phf2. After ChREBP silencing, hepatocytes were incubated 24 h with either 5 or 25 mM glucose. **f** Representative western blot analysis of glycolytic and lipogenic enzymes (*n* = 3). **g** *De novo* lipogenic rate measured *in vitro* by tracing newly FA from ^{14}C-glucose for 2 h and hepatocyte TG content (*n* = 3). All error bars represent mean ± SEM. Statistical analyses were made using Anova, followed by Bonferonni's test. **P* < 0.05 USi 25 mM compared to USi 5 mM, ***P* < 0.01 Phf2i 25 mM compared to USi 25 mM, #*P* < 0.01 USi 25 mM compared to USi 5 mM, $ *P* < 0.01 Phf2/USi 25 mM compared to USi 25 mM, £ *P* < 0.01 Phf2/ChREBPi 25 mM compared to Phf2/USi 25 mM.

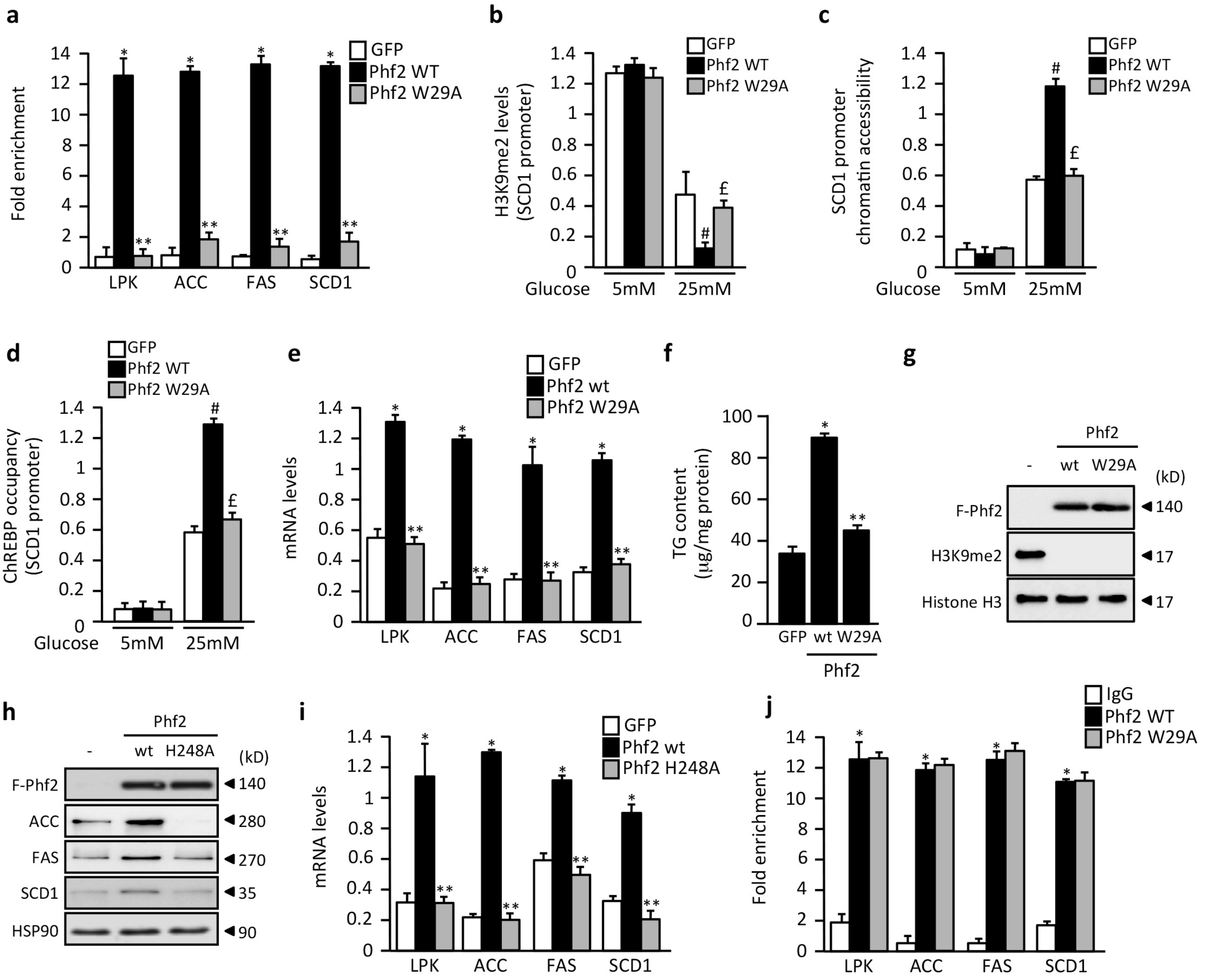

Supplementary Fig. 6 Phf2 H3K9me2 histone demethylase activity is essential to regulate ChREBP function. **(a-d)** FLAG-tagged wild-type (wt) or W29A mutant of Phf2 were overexpressed in primary cultured hepatocytes. After Phf2 overexpression, hepatocytes were incubated in the presence of 5 or 25 mM glucose for 24 h. **a** ChIP experiments showing the recruitment of FLAG-tagged Phf2 (WT or W29A) at the ChoRE-containing region of glycolytic and lipogenic gene promoter (*n* = 3). **b** ChIP experiments for H3K9me2 at the SCD1 promoter (*n* = 3). **c** Chromatin accessibility at the SCD1 promoter (*n* = 3). **d** ChIP experiments showing the recruitment of ChREBP at the SCD1 promoter (*n* = 3). **e** Expression of glycolytic and lipogenic genes (*n* = 3). **f** TG contents (*n* = 3). **g** Demethylase activity of either FLAG-tagged wild-type (wt) or W29A mutant of Phf2, immunoprecipitated (IP) from 293T cell lysates, was assessed using recombinant H3K9me2 methylated histone as substrate (n=2). **(h-j)** FLAG-tagged wild-type (wt) or H248A mutant of Phf2 were overexpressed in primary cultured hepatocytes for 24 h. **h** Western blot analysis against glycolytic and lipogenic enzymes (*n* = 3). **i** Expression of glycolytic and lipogenic genes (*n* = 3). **j** ChIP experiments showing the recruitment of FLAG-tagged Phf2 (WT or H248A) at the ChoRE-containing region of glycolytic and lipogenic gene promoter (*n* = 3). All error bars represent mean ± SEM. Statistical analyses were made using unpaired Anova, followed by Bonferonni's test. *$P < 0.01$ Phf2 (wt) compared to GFP, **$P < 0.01$ Phf2 (wt) compared to Phf2 (W29A or H248A), #$P < 0.01$ Phf2 (wt) 25 mM glucose compared to GFP 25 mM glucose, £$P < 0.01$ Phf2 (wt) 25 mM glucose compared to Phf2 (W29A) 25 mM glucose.

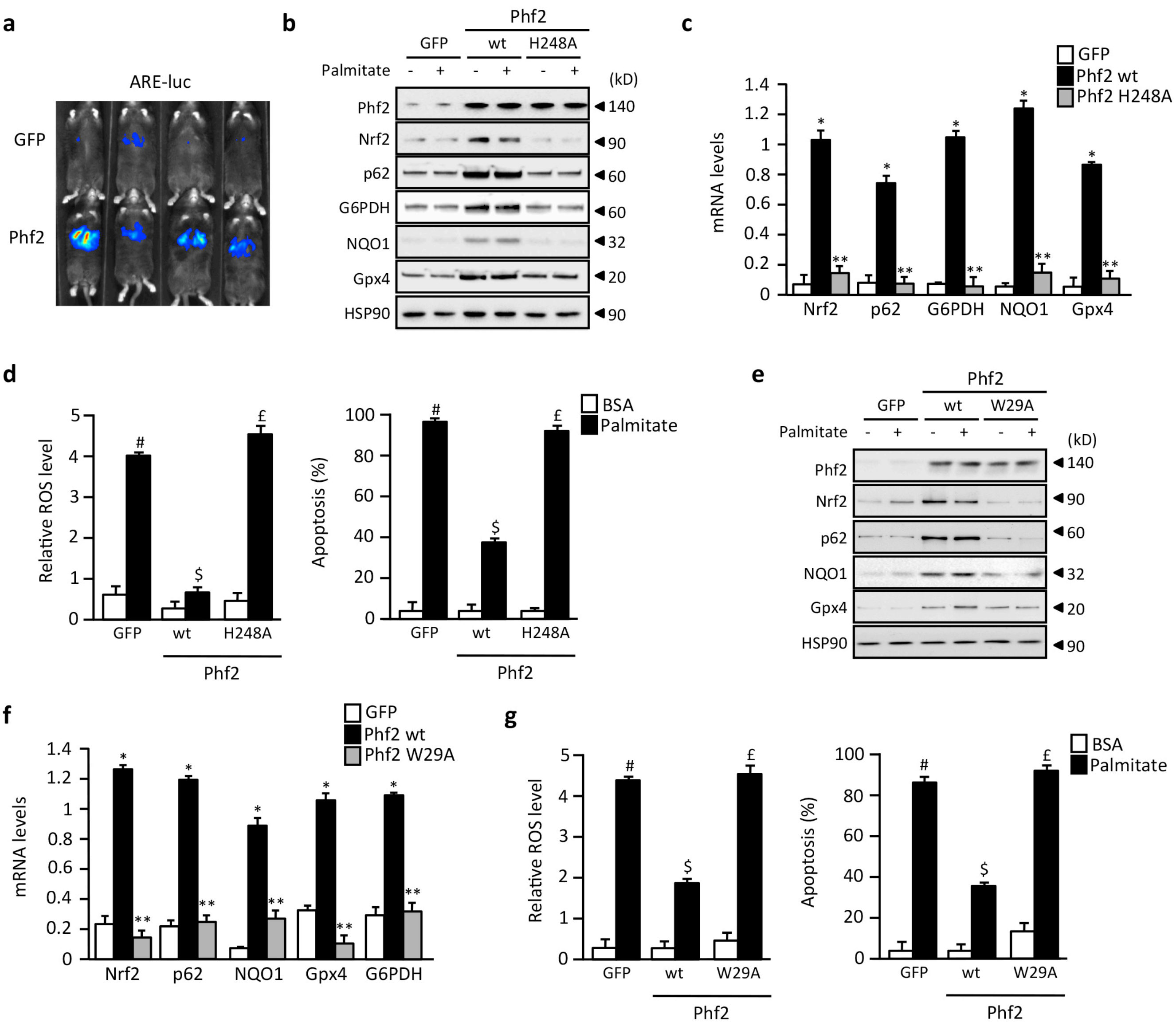

Supplementary Fig. 7 Phf2 H3K9me2 histone demethylase activity is essential to mediate its effects against oxidative stress. **(A)** *In vivo* imaging measurement of Nrf2 activity on the ARE-luciferase (ARE-luc) construct after liver-specific Phf2 overexpression (*n* = 10 mice per group). **(B-D)** FLAG-tagged wild-type (wt) or H248A mutant of Phf2 were overexpressed in primary cultured hepatocytes. After Phf2 overexpression, hepatocytes were incubated in the presence of palmitate (480 μM) for 24 h. **B,** Representative western blots showing proteins involved in the defense against oxidative stress (*n* = 3). **C,** Relative expression of anti-oxidative genes (*n* = 3). **D,** Relative ROS levels and measurement of hepatocyte apoptosis (*n* = 3). **(E-G)** FLAG-tagged wild-type (wt) or W29A mutant of Phf2 were overexpressed in primary cultured hepatocytes. After Phf2 overexpression, hepatocytes were incubated in the presence of palmitate (480 μM) for 24 h. **E,** Representative western blots showing proteins involved in the defense against oxidative stress (*n* = 3). **F,** Relative expression of anti-oxidative genes (*n* = 3). **G,** Relative ROS levels and measurement of hepatocyte apoptosis (*n* = 3). All error bars represent mean ± SEM. Statistical analyses were made Anova, followed by Bonferonni's test. **P* < 0.01 Phf2 (wt) compared to GFP, ***P* < 0.01 Phf2 (wt) compared to Phf2 (W29A or H248A), #*P* < 0.01 GFP palmitate compared to GFP BSA, $*P* < 0.01 Phf2 (wt) palmitate compared to GFP palmitate, £*P* < 0.01 Phf2 (wt) palmitate compared to Phf2 (H248A or W29A) palmitate.

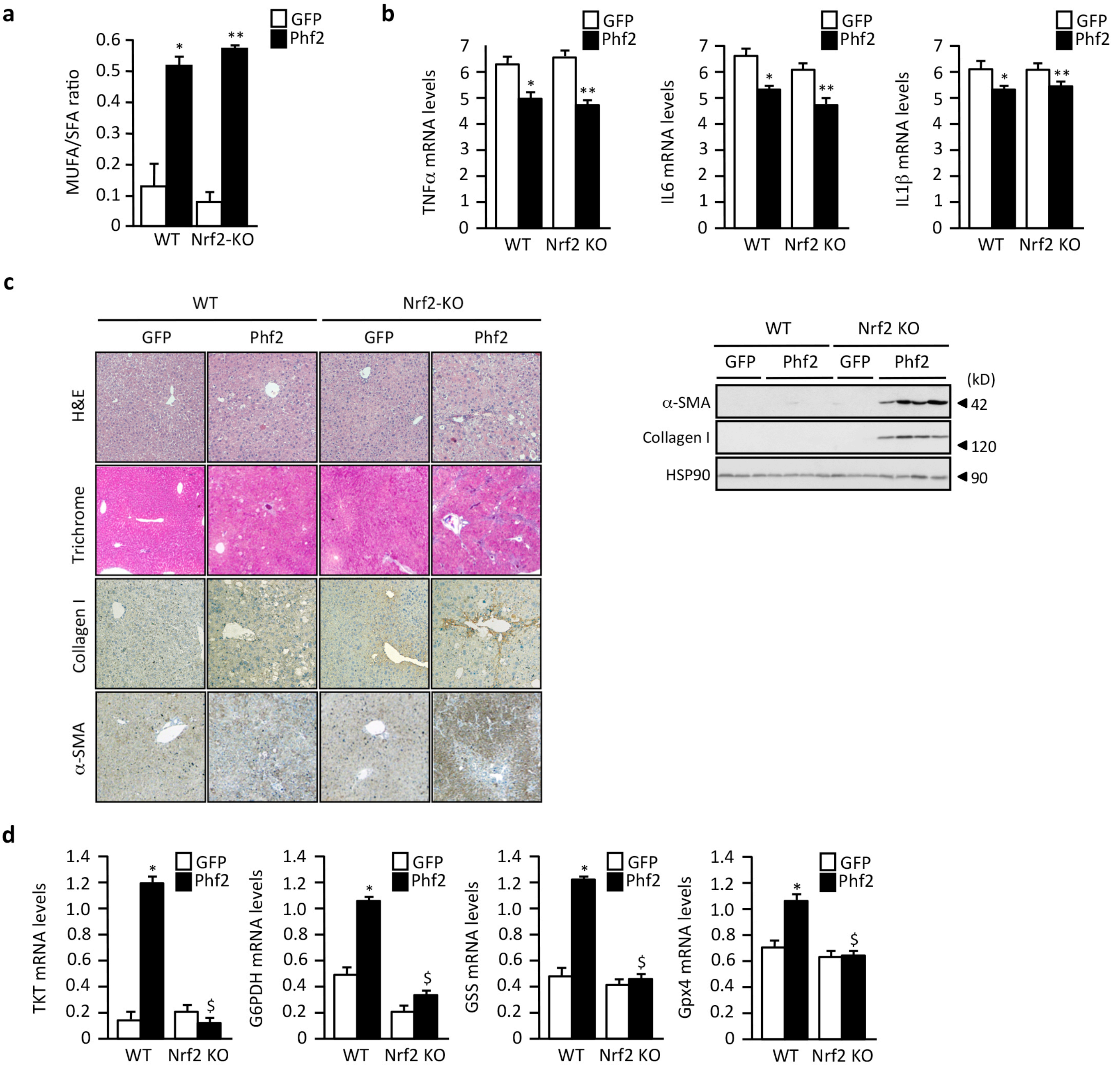

Supplementary Fig. 8 Phf2-driven Nrf2 activation protects the liver from the pro-fibrogenic response during NAFLD development. Wild Type (WT) and Nrf2 knockout (Nrf2-KO) mice were injected with adenovirus overexpressing either GFP or Phf2 and were studied 3 weeks later in the fed state. **a** MUFA/SFA ratio reflecting SCD1 activity (n = 10 per group). **b** Relative expression of liver pro-inflammatory genes (n = 10 per group). **c** (left) Liver sections, stained with hematoxylin and eosin (H&E), trichrome masson, α-SMA and collagen I are shown. (right) Representative western blot analysis of α-SMA and collagen I in the liver (n = 10 per group). **d** Relative expression of liver TKT, G6PDH, GSS and Gpx4 (n = 10 per group). All error bars represent mean ± SEM. Statistical analyses were made using Anova, followed by Bonferonni's test. *P < 0.01 WT/GFP compared to WT/Phf2, **P < 0.01 Nrf2KO/GFP compared to Nrf2KO/Phf2, $ P < 0.01 Nrf2KO/Phf2 compared to WT/Phf2.

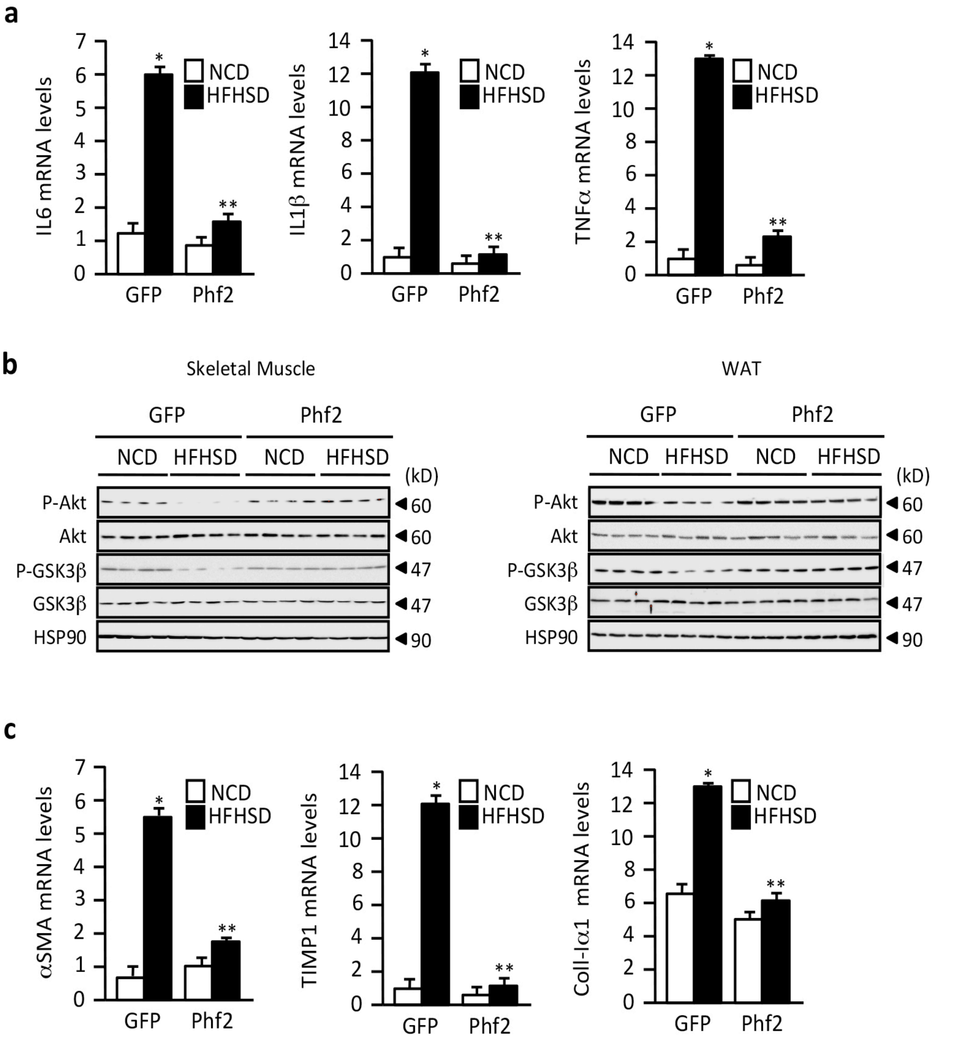

Supplementary Fig. 9 Phf2 protects the liver from HFHSD-induced fibrogenesis. GFP or Phf2 were overexpressed through AAV strategy in the liver of C57Bl/6J mice fed with either a normal chow diet (NCD) or with a high fat and high sucrose diet (HFHSD) for 16 weeks. Mice were then studied in the fed state. **a** Relative expression of liver pro-inflammatory genes (n = 15 mice per group). **b** Western blot analysis of the PI3K/Akt signaling pathway in white adipose tissue and skeletal muscle (n = 10 per group). **c** Expression of pro-fibrogenic genes (n = 15 mice per group). All error bars represent mean ± SEM. Statistical analyses were made using Anova, followed by Bonferonni's test. *P < 0.01 HFHSD/GFP compared to NCD/GFP, **P < 0.01 HFHSD/Phf2 compared to HFHSD/GFP.

Supplementary Tables

Supplementary Table 1

Metabolic characteristics of liver-specific Phf2 overexpressing mice

Mice, injected with either GFP or Phf2 overexpressing adenovirus, were studied 3 weeks later in the fed state. (n=15 per group). Statistical analyses were made using unpaired t-test.
* *P* < 0.05 Phf2 compared to GFP

	GFP	Phf2
Food intake (g/day)	1.74 ± 0.11	2.37 ± 0.08*
Body weight (g)	28.1 ± 1.04	23.4 ± 1.18*
Body weight gain (g)	7.4 ± 1.12	1.3 ± 1.56*
Liver weight (g)	0.81 ± 0.04	1.87 ± 0.07*
Epididimal white adipose tissue weight (g)	0.523 ± 0.07	0.294 ± 0.05*
Post prandial glycemia (mg/dl)	155 ± 5.4	122 ± 2.7*
Post prandial insulinema (ng/ml)	12.32 ± 0.7	10.02 ± 0.5*
Plasma Alanine aminotransferase (units/l)	30 ± 5.4	28.5 ± 3.5
Plasma Aspartate aminotransferase (units/l)	12.9 ± 3.1	10.7 ± 5.7
Plasma triglycerides (mg/dl)	0.98 ± 0.04	1.74 ± 0.05*
Plasma free fatty acids (mmol/l)	1.45 ± 0.18	1.28 ± 0.08
Liver Triglycerides (mg /g of liver)	30.25 ± 1.74	165.23 ± 3.85*
Liver DAG (mg /g of liver)	0.58 ± 0.04	1.25 ± 0.08*
Liver ceramides (mmol / mg of liver)	17.51 ± 2.78	15.23 ± 1.56
Liver cholesterol esters (mg /g of liver)	0.22 ± 0.02	0.97 ± 0.05*
FA uptake (nmol/h/mg liver)	155.75 ± 6.12	315.21 ± 8.37*
FA esterification (nmol/h/mg liver)	13.42 ± 1.02	25.01 ± 2.24*
Lipogenesis (nmol/h/mg liver)	18.14 ± 3.29	27.98 ± 2.18*
Liver Glycogen (mg/g of liver)	7.51 ± 1.14	3.87 ± 1.07*
Liver Reduced glutathione (GSH)	0.78 ± 0.02	1.57 ± 0.05*
Liver Oxidized glutathione (GSSR)	0.87 ± 0.17	1.01 ± 0.14
Liver GSH/GSSR ratio	0.89 ± 0.03	1.55 ± 0.04*
Skeletal muscle glycogen (mg / g)	7.5 ± 0.2	7.8 ± 0.4

Supplementary Table 2

Metabolic characteristics of WT and Nrf2-KO mice overexpressing Phf2

GFP or Phf2 were overexpressed in the liver of Wild Type (WT) and Nrf2 knockout (Nrf2-KO) mice.
Mice were studied 3 weeks later in the fed state.
(n=12 per group). Statistical analyses were made using Anova followed by Bonferonni's test.
* *P* < 0.05 Phf2 (WT or Nrf2-KO) compared to GFP (WT or NRF2-KO)

	WT		Nrf2-KO	
	GFP	Phf2	GFP	Phf2
Glycemia (mg/dl)	145.24 ± 4.12	107.18 ± 1.36	158.66 ± 7.89	128.10 ± 6.33
Body weight (g)	29 ± 0.94	25.3 ± 1.01	25.6 ± 1.14	24.6 ± 0.87
Body weight gain (g)	6.3 ± 1.23	1.1 ± 1.47*	6.4 ± 1.24	1.4 ± 1.26*
Liver weight (g)	1.47 ± 0.10	2.37 ± 0.06*	1.03 ± 0.02	2.25 ± 0.04
Epididimal white adipose tissue weight (g)	0.471 ± 0.02	0.238 ± 0.02*	0.466 ± 0.05	0.266 ± 0.03*
Plasma Alanine aminotransferase (units/l)	42.25 ± 2.72	55.78 ± 3.38	47.56 ± 7.19	127.84 ± 6.47*
Plasma Aspartate aminotransferase (units/l)	10.13 ± 1.43	18.74 ± 2.77	14.72 ± 4.31	33.98 ± 4.11*
Plasma triglycerides (mg/dl)	0.88 ± 0.09	1.52 ± 0.11*	1.12 ± 0.07	1.77 ± 0.14*
Plasma free fatty acids (mmol/l)	1.24 ± 0.14	1.38 ± 0.45	1.25 ± 0.27	1.36 ± 0.17
Liver triglycerides (mg/g)	45.20 ± 5.19	300.14 ± 10.28*	45.75 ± 7.37	320.48 ± 17.34*

Supplementary Table 3

Characteristics of liver-specific GFP and Phf2 overexpressing mice fed on NCD or HFHSD diets for 16 weeks

GFP or Phf2 were overexpressed through AAV strategy in the liver of seven-week-old male C57Bl/6J mice.
Mice were then fed with either a chow diet (NCD) or with a high fat and high sucrose diet (HFHSD) for 16 weeks.
Mice were studied in the fed state.
(n=15 per group). Statistical analyses were made using Anova followed by Bonferonni's test.
* P < 0.05 GFP NCD compared to GFP HFHSD, * P < 0.05 GFP HFHSD compared to Phf2 HFHSD, # P < 0.05 GFP NCD compared to Phf2 NCD

	GFP		Phf2	
	NCD	HFHSD	NCD	HFHSD
Food intake (g/day)	1.86 ± 0.05	2.21 ± 0.02	2.28 ± 0.03#	2.90 ± 0.09**
Body weight gain (g)	7.16 ± 1.05	24.91 ± 2.32*	4.28 ± 1.11#	6.50 ± 1.76**
Epididimal white adipose tissue weight (g)	1.24 ± 0.12	2.53 ± 0.20*	0.61 ± 0.08#	1.52 ± 0.23**
Serum insulin (ng/ml)	0.51 ± 0.10	4.41 ± 1.54*	0.31 ± 0.21	0.76 ± 0.45**
Glycemia (mg/dl)	139.76 ± 15.03	180.38 ± 17.44*	124.54 ± 8.14	129.76 ± 15.72**
Plasma Aspartate aminotransferase (units/l)	10.22 ± 1.15	48.79 ± 2.88*	10.72 ± 3.32	12.90 ± 2.08**
Plasma Alanine aminotransferase (units/l)	39.67 ± 1.48	141.71 ± 3.72*	33.57 ± 2.64	44.24 ± 3.51**
Plasma free fatty acids (mmol/l)	0.77 ± 0.04	0.67 ± 0.13	1.985 ± 0.14	0.81 ± 0.05
Plasma triglycerides (mg/dl)	0.56 ± 0.09	0.86 ± 0.14*	0.54 ± 0.08	0.76 ± 0.22
Reduced glutathione (nmol/mg protein)	25.12 ± 1.02	15.08 ± 2.05*	45.26 ± 1.47#	48.75 ± 4.35**
Oxidized glutathione (nmol/mg protein)	0.85 ± 0.05	2.11 ± 0.31*	0.77 ± 0.02	0.81 ± 0.07**

Supplementary Table 4

Clinical and biochemical characteristics of lean and obese patients

(n=10 per group). Statistical analyses were made using Anova followed by Bonferonni's test.
* P < 0.05 compared to control

	NoFL	Steatosis	NASH
Age	47.3 ± 12.87	38.1 ± 10.04	53.4 ± 8.94
BMI Kg/m^2	23.8 ± 1.78	48.5 ± 5.75*	44.7 ± 4.35*
Fasted glycemia (mmol/l)	4.4 ± 0.61	4.7 ± 0.26	10.8 ± 2.97*
Glycemia at 120 min after HPO (mmol/l)	5.2 ± 0.84	5.7 ± 0.97	18.6 ± 3.75*
Fasted insulinemia (IU/l)	5.9 ± 1.68	12.6 ± 5.04*	50.8 ± 10.72*
HbA1c (%)	5.51 ± 0.73	5.40 ± 0.12	9.58 ± 0.42*
HOMA-IR	0.7 ± 0.33	1.5 ± 0.63	6.6 ± 1.43*
Alanine aminotransferase (IU/l)	22.1 ± 5.17	28.6 ± 3.55	55.8 ± 10.11*
Aspartate aminotransferase (IU/l)	23.4 ± 12.69	32.2 ± 18.56	112.2 ± 8.77*
ASAT/ALAT ratio	1.05 ± 0.17	1.12 ±0.72	2.01 ±0.38*
γ-GT (IU/l)	48.1 ± 5.04	25.4 ± 7.14	98.8 ±10.06*
Plasma triglycerides (mg/dl)	1.2 ± 0.40	1.69 ± 0.65	2.43 ± 1.03*
% of hepatocytes with steatosis	4.4 ± 0.61	62 ± 9.18*	65 ± 16.74*
NAS score	0.4 ± 0.53	2.5 ± 0.11*	6.4 ± 0.32*

Supplementary Table 5:

Histological scoring used for NAS and fibrosis determination

(n=10 per group)

	NoFL	Steatosis	NASH
Steatosis			
0 (n)	6	0	0
1 (n)	4	0	0
2 (n)	0	5	4
3 (n)	0	5	6
Hepatocyte ballooning			
0 (n)	10	10	0
1 (n)	0	0	3
2 (n)	0	0	7
Lobular inflammation			
0 (n)	10	10	0
1 (n)	0	0	3
2 (n)	0	0	7
3 (n)	0	0	0
Fibrosis Stage			
0 (n)	10	6	0
1 (n)	0	3	0
1a (n)	0	1	0
1b (n)	0	0	0
1c (n)	0	0	0
2 (n)	0	0	4
3 (n)	0	0	6
4 (n)	0	0	0
